# Repeated administration of an acetylcholinesterase inhibitor attenuates nicotine taking in rats and smoking behavior in human smokers

**DOI:** 10.1038/tp.2015.209

**Published:** 2016-01-19

**Authors:** R L Ashare, B A Kimmey, L E Rupprecht, M E Bowers, M R Hayes, H D Schmidt

**Affiliations:** 1 Center for Interdisciplinary Research on Nicotine Addiction, Department of Psychiatry, Perelman School of Medicine, University of Pennsylvania, Philadelphia, PA, USA; 2 Center for Neurobiology and Behavior, Department of Psychiatry, Perelman School of Medicine, University of Pennsylvania, Philadelphia, PA, USA; 3 Translational Neuroscience Program, Department of Psychiatry, Perelman School of Medicine, University of Pennsylvania, Philadelphia, PA, USA; 4 Department of Biobehavioral Health Sciences, School of Nursing, University of Pennsylvania, Philadelphia, PA, USA

## Abstract

Tobacco smoking remains the leading cause of preventable death worldwide and current smoking cessation medications have limited efficacy. Thus, there is a clear need for translational research focused on identifying novel pharmacotherapies for nicotine addiction. Our previous studies demonstrated that acute administration of an acetylcholinesterase inhibitor (AChEI) attenuates nicotine taking and seeking in rats and suggest that AChEIs could be repurposed for smoking cessation. Here, we expand upon these findings with experiments designed to determine the effects of repeated AChEI administration on voluntary nicotine taking in rats as well as smoking behavior in human smokers. Rats were trained to self-administer intravenous infusions of nicotine (0.03 mg kg^−1^ per 0.59 ml) on a fixed-ratio-5 schedule of reinforcement. Once rats maintained stable nicotine taking, galantamine or donepezil was administered before 10 consecutive daily nicotine self-administration sessions. Repeated administration of 5.0 mg kg^−1^ galantamine and 3.0 mg kg^−1^ donepezil attenuated nicotine self-administration in rats. These effects were reinforcer-specific and not due to adverse malaise-like effects of drug treatment as repeated galantamine and donepezil administration had no effects on sucrose self-administration, *ad libitum* food intake and pica. The effects of repeated galantamine (versus placebo) on cigarette smoking were also tested in human treatment-seeking smokers. Two weeks of daily galantamine treatment (8.0 mg (week 1) and 16.0 mg (week 2)) significantly reduced smoking rate as well as smoking satisfaction and reward compared with placebo. This translational study indicates that repeated AChEI administration reduces nicotine reinforcement in rats and smoking behavior in humans at doses not associated with tolerance and/or adverse effects.

## Introduction

Cigarette smoking remains the leading cause of preventable disease and death in the United States, accounting for one of every five deaths annually.^1^ Although there are FDA-approved pharmacotherapies available for nicotine dependence, including nicotine replacement therapies, bupropion and varenicline, relapse rates remain high—approximately 75% of smokers relapse within 6 months.^2, 3^ Thus, there is a clear need to develop novel medications for nicotine addiction. Repurposing medications that have been ‘de-risked' through prior development bypasses many barriers associated with typical drug discovery strategies (that is, cost and duration).^4^

Acetylcholinesterase inhibitors (AChEIs), which are FDA-approved for treating cognitive deficits associated with Alzheimer's disease,^5^ have recently been proposed as potential treatments for drug addiction, including nicotine dependence.^6, 7^ AChEIs increase extracellular levels of acetylcholine in the brain and augment cholinergic transmission through inhibition of acetylcholinesterase, a catabolic enzyme responsible for metabolizing acetylcholine in the synapse.^8, 9^ Recently, we showed that acute administration of the AChEIs galantamine^10^ and donepezil^11^ attenuated nicotine self-administration in rats. These preclinical findings are provocative and suggest that AChEIs could be repurposed as pharmacotherapies for smoking cessation.

Recent clinical studies have begun to explore the effects of AChEIs on smoking behavior. Preliminary studies with galantamine in alcohol-dependent, methamphetamine-dependent and schizophrenic smokers show that it is well tolerated but provide mixed results for efficacy.^12, 13, 14, 15^ Moreover, donepezil treatment modestly improved cognition, but had no effect on smoking behavior.^16^ These contradictory findings are likely due to study limitations including small sample sizes, genetic variability, poly-drug use and comorbid neuropsychiatric disorders. In contrast, recent evidence indicates that galantamine reduces the subjective effects of an acute intravenous nicotine infusion in healthy human smokers.^17^ However, the efficacy of AChEIs for smoking cessation remains to be defined in healthy, treatment-seeking smokers without comorbidities.

Successful development of novel smoking cessation medications requires translational studies that advance and accelerate preclinical findings into pilot clinical trials.^18, 19^ Although studies of acute AChEI administration on nicotine taking in rats are informative,^10, 11^ these studies are limited in their translation to clinical research primarily because that they do not model the repeated dosing regimen that is likely required to treat human smokers attempting to quit. Therefore, studies of repeated AChEI administration on nicotine self-administration are needed.

The overarching goal of this study was to screen the potential efficacy of repeated AChEI administration in rat and human models of nicotine addiction to provide sufficient rationale for large-scale clinical trials of AChEIs in human smokers. As a logical extension of our previous studies, we examined the effects of repeated AChEI administration on nicotine self-administration in rats. To translate findings from preclinical to clinical research, the effects of repeated galantamine on smoking behavior and the subjective effects of smoking were investigated in treatment-seeking human smokers. The reported adverse effects of AChEIs are similar to other cholinergic agents and include malaise symptoms, such as nausea and vomiting.^20, 21^ Therefore, the effects of repeated AChEI administration on *ad libitum* food intake and pica, an animal model that is used to assess rodent consumption of non-nutritive materials (for example, kaolin clay) in response to nauseating agents,^22^ were tested in separate cohorts of rats. Side effects of galantamine in human smokers were also assessed. We hypothesized that repeated AChEI administration would reduce nicotine self-administration in rats and smoking rate in humans at doses associated with minimal adverse effects.

## Materials and methods

### Rat studies

Details regarding animals, housing, surgery and acquisition of nicotine and sucrose self-administration can be found in Supplementary Materials and Methods and are identical to those described in our previously published studies.^10, 11, 23^

#### Nicotine self-administration following repeated AChEI administration

The effects of repeated AChEI administration on nicotine taking were examined in separate cohorts of rats that acquired stable nicotine self-administration on a fixed-ratio-5 schedule of reinforcement. The rats were randomly divided into different treatment groups and a between-subjects design was used to test the effects of repeated galantamine and donepezil on nicotine taking. In all the experiments, the rats were pretreated with galantamine (0, 0.5 or 5.0 mg kg^−1^, intraperitoneally) or donepezil (0, 0.1, 1.0 or 3.0 mg kg^−1^, intraperitoneally) 20 min before the beginning of the operant test sessions. Rats were pretreated with galantamine or donepezil daily for 10 consecutive test days similar to previous studies of repeated drug administration on nicotine self-administration.^24, 25^ The time course of administration was chosen on the basis of previously published reports of galantamine^26, 27^ and donepezil.^28, 29^ Doses were selected on the basis of our previously published reports demonstrating that acute administration of galantamine^10^ and donepezil^11^ dose-dependently attenuated nicotine taking and seeking in rats.

#### Sucrose self-administration following repeated AChEI administration

Once the rats acquired stable sucrose self-administration on a fixed-ratio-5 schedule of reinforcement, they were randomly divided into different treatment groups. The effects of repeated AChEI administration on sucrose taking were tested in separate cohorts of rats using a between-subjects design. The rats were pretreated daily with galantamine (0, 0.5 or 5.0 mg kg^−1^, intraperitoneally) or donepezil (0, 0.1, 1.0 or 3.0 mg kg^−1^, intraperitoneally) 20 min before the beginning of the sucrose self-administration test sessions for 10 consecutive test days.

#### Pica and *ad libitum* food intake

Separate cohorts of rats were pretreated daily for 10 consecutive days with galantamine (0, 0.5 and 5.0 mg kg^−1^, intraperitoneally) or donepezil (0, 0.1, 1.0 and 3.0 mg kg^−1^, intraperitoneally) 20 min before testing. Pre-weighed standard rodent chow (Purina 5001) and kaolin pellets were placed in each cage at the onset of the dark cycle. Cumulative chow and kaolin intake (±0.1 g) were recorded 24 h following onset of the dark cycle. The changes in body weight were also recorded over the 24 h testing session. A between-subjects design was used to test the potential effects of repeated AChEI administration on kaolin intake and *ad libitum* food consumption. See Supplementary Materials and Methods for more details.

### Human studies

#### Participants

Male and female treatment-seeking smokers, ages 18–60 years old who smoked at least 10 cigarettes per day for the previous 6 months were recruited from the community via advertisements. Smokers had to rate their confidence in making a quit attempt in the next 6 months as 50 or higher (on a scale from 0 to 100) to be eligible. Participants provided written informed consent and completed an in-person eligibility screen including a urine drug screen, a breath alcohol test and an expired breath carbon monoxide (CO) reading to confirm smoking status (at least 10 p.p.m.); women completed a urine pregnancy test. Exclusion criteria and participant characteristics can be found in the Supplementary Materials and Methods (Supplementary Table S1).

#### Study design and procedures

The University of Pennsylvania Institutional Review Board approved all the study procedures. This was a randomized double-blind, between-subjects, placebo-controlled study comparing 3 weeks galantamine treatment to placebo. The focus of the current paper is on smoking behavior and subjective effects of galantamine versus placebo during the pre-quit period (that is, first two weeks of medication). Additional details regarding the study design can be found in the Supplementary Materials and Methods. After eligibility was confirmed, smokers completed a 2-week run-up period during which they were treated with placebo or 8 mg galantamine daily for the first week and placebo or 16 mg galantamine (daily) during the second week. Following the intake, participants completed a 1.5-h baseline visit to assess smoking rate, side effects and subjective effects of smoking. After 1 week on medication, participants attended a brief 20- min observation (week 1) visit to assess side effects. At the week 2 visit, participants completed the same measures as the baseline visit.

#### Smoking outcomes

The primary smoking outcomes were self-reported cigarettes per day and CO. At each visit, a standard timeline follow-back method was used to assess self-reported smoking rate,^30^ and expired breath CO samples were collected as biochemical verification of smoking behavior within 10 min of the start of each visit.

#### Secondary outcomes

As in our prior work,^16^ side effects were monitored using a 37-item side-effect checklist with common side effects of galantamine (for example, nausea, vomiting) rated on a four-point scale (0=none, 1=mild, 2=moderate, 3=severe). A summary score was calculated for each visit. Because pica was used to measure nausea/malaise in rodents, nausea was examined separately. A dichotomous variable was created such that 0 indicated no nausea and 1 indicated any rating of nausea greater than 0. The 11-item Cigarette Effects Scale^31^ assessed satisfaction and psychological reward associated with smoking. Items were rated on a Likert scale from 1 (not at all) to 7 (extremely). All measures were assessed at the baseline, week 1 and week 2 visits.

#### Covariates and exploratory outcomes

Smoking history (for example, cigarettes per day, age smoking initiation) and the Fagerström Test for Nicotine Dependence;^32^ were assessed at the eligibility screen. Craving, withdrawal and affect were considered exploratory outcomes because subjects were still smoking during the 2-week assessment and therefore we expected few changes in these measures. Craving was assessed with the 10-item Questionnaire of Smoking Urges-Brief.^33^ The Questionnaire of Smoking Urges-Brief contains two subscales (anticipation of reward, relief from negative affect) and items are rated on a seven-point Likert scale ranging from 1 (strongly disagree) to 7 (strongly agree). The Minnesota Nicotine Withdrawal Scale was used to assess nicotine withdrawal.^34, 35^ Withdrawal symptoms were rated on a scale from 0 (none) to 4 (severe) and a summary score was calculated. Affect was assessed using the 20-item Positive and Negative Affect Schedule,^36^ which measures two generally orthogonal dimensions of affect: positive affect (10 items, for example, enthusiastic, strong) and negative affect (10 items, for example, distressed, upset). Items are rated on a scale from 1 (not at all) to 5 (extremely).

#### Drugs

(−)Nicotine hydrogen tartrate salt (Sigma Aldrich, St. Louis, MO, USA) was dissolved in sterile 0.9% saline (pH was adjusted to 7.4±0.5 with sodium hydroxide). Galantamine hydrobromide and donepezil hydrochloride (Tocris, Ellisville, MS, USA) were dissolved in sterile 0.9% saline. Nicotine doses are reported as freebase concentrations whereas galantamine and donepezil concentrations are reported as their salt concentrations. Investigational Drug Services at the University of Pennsylvania packaged galantamine hydrobromide extended release and placebo in matching capsules to ensure double-blind testing conditions. The dosing regimen consisted of an initial 1-week 8 mg galantamine run-up (daily). During week 2, the dose was titrated to the recommended 16 mg daily dose.^37^ Participants were instructed to take the study medication at the same time each day, preferably in the morning and with food. Medication adherence was assessed by participant self-report and confirmed with reconciliation of medication dispensed versus returned. Adherence was calculated as percentage of pills consumed (out of 14).

### Statistical analyses

Total lever responses, total nicotine infusions and total sucrose pellets self-administered during all AChEI experiments were analyzed with two-way analyses of variance with repeated measures over time. Total chow and kaolin intake as well as changes in body weight were also analyzed with two-way analyses of variance with repeated measures over time. Pairwise comparisons were made with Bonferroni correction (*P*<0.05). For the human study, univariate statistics were generated to describe the demographics and smoking characteristics, and chi-square or *t*-tests assessed baseline group differences. Smoking rate, CO levels, side effects, smoking satisfaction and smoking reward were analyzed with maximum-likelihood regression models with visit (baseline, week 1, week 2) as a within-subjects repeated measure factor and group (galantamine versus placebo) as a between-subject factor. The group × visit interaction was tested and Bonferroni corrected for five outcomes (*P<*0.01). Planned contrasts tested the linear effect (that is, baseline versus week 2). For the dichotomous nausea variable, chi-square analyses were conducted to compare group at each time point separately. Because craving, withdrawal and affect were exploratory outcomes, no correction for multiple outcomes was made (*P*=0.05). Effect sizes for group differences are reported using Cohen's *d*. All models controlled for nicotine-dependence level.

## Results

### Rat studies

#### Repeated galantamine administration dose-dependently attenuated nicotine, but not sucrose, self-administration in rats

**Figure 1:**
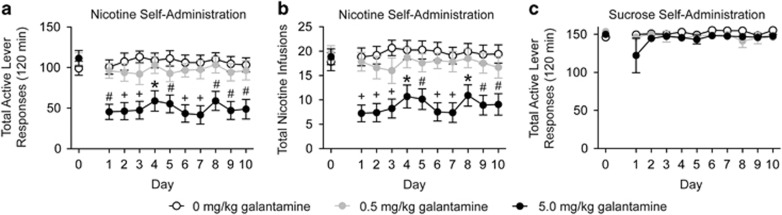
Repeated administration of galantamine dose-dependently attenuated nicotine, but not sucrose, self-administration in rats. Galantamine pretreatment significantly attenuated total active lever responses (**a**) and total nicotine infusions (**b**) in rats stably self-administering nicotine on an FR5 schedule of reinforcement (*n*=12, 0 mg kg^−1^ galantamine; *n*=13, 0.5 mg kg^−1^ galantamine; *n*=12, 5.0 mg kg^−1^ galantamine). *P<0.05, ^#^*P*<0.01, ^+^*P*<0.001 between vehicle and 5.0 mg kg^−1^ galantamine treatments. (**c**) There were no effects of repeated galantamine administration on sucrose self-administration (*n*=5, 0 mg kg^−1^ galantamine; *n*=5, 0.5 mg kg^−1^ galantamine; *n*=6, 5.0 mg kg^−1^ galantamine). FR5, fixed-ratio-5.

First, we tested the effects of repeated galantamine administration on nicotine taking in rats. The rats that maintained stable responding for nicotine were randomly assigned to one of three treatments groups. The rats that were designated to received vehicle (*n*=12), 0.5 mg kg^−1^ galantamine (*n*=13) or 5.0 mg kg^−1^ galantamine (*n*=12) self-administered the same number of nicotine infusions (mean±s.e.m.) before the treatment phase of the experiment: 17.83±1.84, 19.61±1.53 and 18.85±1.62, respectively (Figure 1b, day 0). Total active lever responses and total nicotine infusions (mean±s.e.m.) for rats pretreated with vehicle or galantamine daily for 10 consecutive days are shown in Figures 1a and b, respectively (days 1–10). The analysis of total active lever data revealed significant main effects of treatment (F(2,34)=10.25, *P*<0.05) and day (F(9,34)=2.19, *P*<0.001). The analysis of total nicotine infusions revealed significant main effects of treatment (F(2,34)=9.99, *P*<0.01) and day (F(9,34)=2.63, *P*<0.001). Subsequent pairwise analyses showed that total active lever responses and total nicotine infusions were significantly decreased in animals pretreated with 5.0 mg kg^−1^ galantamine when compared with saline-treated controls for each day of the 10-day treatment phase (Bonferroni, *P*<0.05). No significant differences were found on inactive lever responding between treatments (F(2,34)=0.75, *P*<0.48; data not shown). Separate cohorts of rats were pretreated with vehicle (*n*=5), 0.5 mg kg^−1^ galantamine (*n*=5) or 5.0 mg kg^−1^ galantamine (*n*=6) daily before 10 consecutive days of sucrose self-administration (Figure 1c). The analysis of these data showed no effect of treatment on sucrose taking (F(2,13)=1.77, *P*<0.21).

#### Repeated galantamine administration does not produce pica or affect food intake in rats

**Figure 2:**
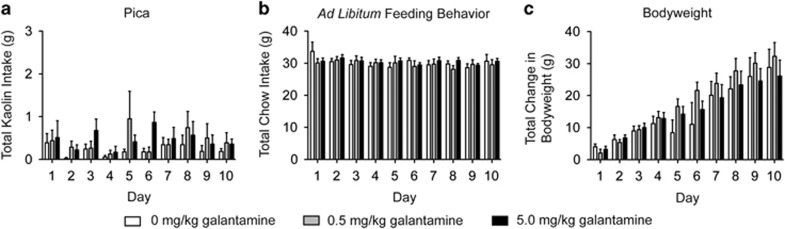
Repeated galantamine administration did not alter pica or feeding behavior in rats. No significant differences in total kaolin (**a**) and chow (**b**) consumed were noted between rats treated with vehicle (*n*=9), 0.5 mg kg^−1^ galantamine (*n*=8) or 5.0 mg kg^−1^ galantamine (*n*=9) for 10 consecutive days. Total change in body weight was also unaffected by repeated galantamine administration compared with controls (**c**).

As galantamine produces adverse effects that could limit patient compliance,^21^ we next determined whether repeated galantamine administration was associated with illness-like behaviors in rats. To determine whether reduced nicotine self-administration in rats pretreated with repeated galantamine is because of malaise-like effects, pica (Figure 2a), *ad libitum* feeding behavior (Figure 2b) and body weight (Figure 2c) were measured in separate cohorts of rats pretreated with vehicle (*n*=9), 0.5 mg kg^−1^ galantamine (*n*=8) and 5.0 mg kg^−1^ galantamine (*n*=9) daily for 10 consecutive days. Analyses of these data showed no significant effects of treatment on pica (F(2,23)=1.12, *P*<0.34), *ad libitum* food intake (F(2,23)=0.12, *P*<0.89) and body weight (F(2,23)=0.46, *P*<0.63).

#### Repeated donepezil administration dose-dependently attenuated nicotine, but not sucrose, self-administration in rats

**Figure 3:**
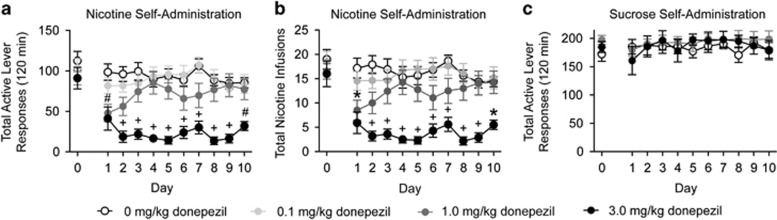
Repeated administration of donepezil dose-dependently attenuated nicotine, but not sucrose, self-administration in rats. Total active lever responses (**a**) and total nicotine infusions (**b**) are shown for rats pretreated with vehicle (*n*=11), 0.1 mg kg^−1^ donepezil (*n*=11), 1.0 mg kg^−1^ donepezil (*n*=12) or 3.0 mg kg^−1^ donepezil (*n*=10) before daily nicotine self-administration sessions. On day 1, total active lever responses and total nicotine infusions were significantly decreased in rats pretreated with 1.0 or 3.0 mg kg^−1^ donepezil compared with vehicle-treated controls (*P<0.05, ^#^*P*<0.01). On days 2–10, total active lever responses and total nicotine infusions were significantly decreased in rats pretreated with 3.0 mg kg^−1^ donepezil compared with vehicle-treated controls (*P<0.05, ^+^*P*<0.001). (**c**) There were no effects of repeated donepezil administration on sucrose self-administration (*n*=8, 0 mg kg^−1^ donepezil; *n*=8, 1.0 mg kg^−1^ donepezil; *n*=9, 3.0 mg kg^−1^ donepezil).

Separate groups of rats were pretreated with vehicle (*n*=11), 0.1 mg kg^−1^ donepezil (*n*=11), 1.0 mg kg^−1^ donepezil (*n*=12) and 3.0 mg kg^−1^ donepezil (*n*=10) daily for 10 consecutive days before nicotine self-administration test sessions. Preceding the treatment phase, these rats self-administered the same total number of nicotine infusions (Figure 3b, day 0; total nicotine infusions (mean±s.e.m.) for the four treatment groups were as follows: vehicle, 19.0±2.02; 0.1 mg kg^−1^ donepezil, 18.54±2.08; 1.0 mg kg^−1^ donepezil, 17.50±1.60; and 3.0 mg kg^−1^ donepezil, 16.50±2.64). Total active lever responses and total nicotine infusions (mean±s.e.m.) for rats pretreated with vehicle and donepezil are shown in Figures 3a and b, respectively (days 1–10). The analysis of active lever data revealed a significant main effect of treatment (F(3,40)=13.46, *P*<0.0001) as well as a significant treatment × day interaction (F(27,40)=2.28, *P*<0.001). The analysis of total nicotine infusions revealed a significant main effect of treatment (F(3,40)=13.46, *P*<0.0001). Subsequent pairwise analyses showed that total active lever responses and total nicotine infusions were significantly different between rats pretreated with 1.0 mg kg^−1^ or 3.0 mg kg^−1^ donepezil and vehicle on day 1 as well as rats pretreated with 3.0 mg kg^−1^ donepezil and vehicle on days 2–10 (Bonferroni, *P*<0.05). There were no significant effects of treatment on inactive lever responding (F(3,40)=1.40, *P*<0.26; data not shown). Separate cohorts of rats were pretreated with vehicle (*n*=8), 1.0 mg kg^−1^ donepezil (*n*=8) or 3.0 mg kg^−1^ donepezil (*n*=9) daily for 10 consecutive days before sucrose self-administration test sessions (Figure 3c). The analysis of these data showed no effect of repeated donepezil treatment on sucrose taking (F(2,22)=0.23, *P*<0.80). Consistent with the galantamine findings in Figure 1, these data indicate that repeated administration of an AChEI is sufficient to attenuate voluntary nicotine taking without producing general behavioral-suppressant effects.

#### Repeated donepezil administration does not produce pica or affect food intake in rats

**Figure 4:**
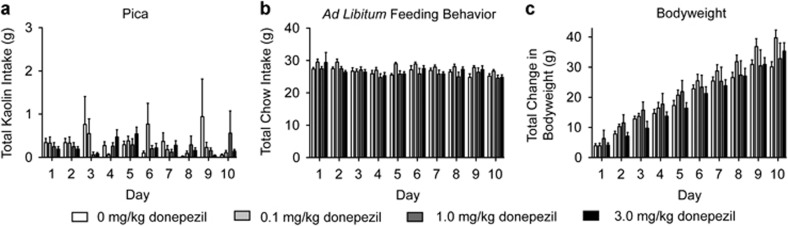
Repeated donepezil administration did not alter pica or feeding behavior in rats. No significant differences in total kaolin (**a**) and chow (**b**) consumed were noted between rats treated with vehicle or donepezil (*n*=10 per treatment) for 10 consecutive days. Total change in body weight was also unaffected by repeated donepezil administration compared with controls (**c**).

Similar to galantamine, donepezil administration also produces nausea and vomiting.^20^ To determine whether repeated donepezil administration produces malaise-like effects at doses that attenuate voluntary nicotine taking, pica (Figure 4a), *ad libitum* feeding behavior (Figure 4b) and body weight (Figure 4c) were measured in separate cohorts of rats pretreated with vehicle, 0.1 mg kg^−1^, 1.0 mg kg^−1^ and 3.0 mg kg^−1^ donepezil (*n*=10 per treatment) for 10 consecutive days. The analyses of these data showed no significant effects of treatment on pica (F(3,36)=0.47, *P*<0.71), *ad libitum* food intake (F(3,36)=1.93, *P*<0.14) and body weight (F(3,36)=0.65, *P*<0.59).

### Human study

#### Participant characteristics

To translate the preclinical findings into clinical research, a randomized pilot clinical trial of the effects of repeated galantamine versus placebo administration on smoking behavior was conducted in human smokers. Overall, 40% (*n*=13) of participants were female and 60% (*n*=20) were African American. On average, participants were 43 years old (s.d.=10.6), had a Shipley IQ score of 103 (s.d.=7.7), reported smoking 14.8 cigarettes per day (s.d.=4.7) for 25 years (s.d.=10.7) and were moderately nicotine dependent (Fagerström Test for Nicotine Dependence mean=4.9, s.d.=2.0). CO levels at the eligibility screen were, on average, 21 p.p.m. (s.d.=8.7). The groups did not differ on any demographic or smoking characteristic (Supplementary Table S1). Medication adherence by pill count was excellent (98.7%) and did not differ by group (placebo mean =98%, range 86–100% galantamine mean =99.5%, range 93–100% *P*=0.2).

#### Repeated galantamine treatment in human smokers reduces smoking behavior

**Figure 5:**
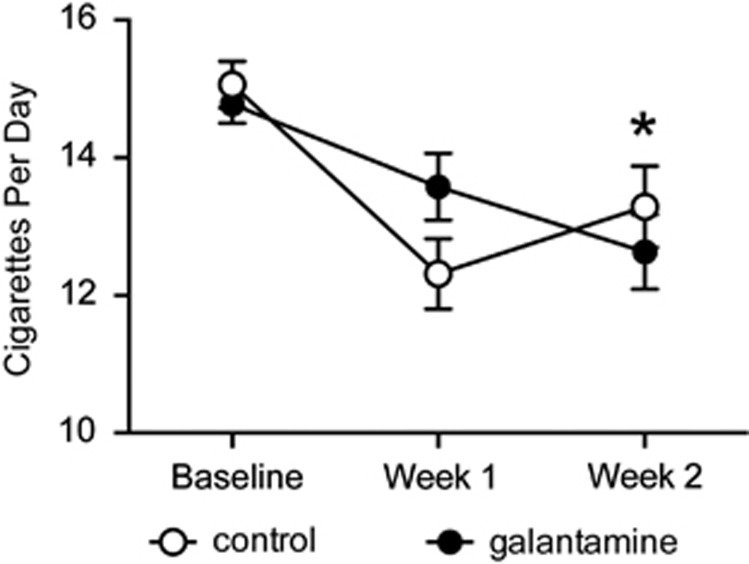
Repeated galantamine treatment in human smokers reduced smoking rate. The total number of cigarettes smoked per day was significantly reduced following 2 weeks of placebo (*n*=18) and galantamine *(n*=15) treatment when compared with baseline smoking rates. Although both groups reported smoking fewer cigarettes from baseline to week 1 (*P*-values <0.001), there was a greater reduction in cigarettes smoked per day in the galantamine group at week 2 when compared with placebo-treated control subjects. *P<0.001.

There were no group differences in cigarettes per day or CO levels at baseline prior to the initiation of treatment (*P*-values *>*0.56; Figure 5 and Supplementary Table S1). Although all subjects reported smoking fewer cigarettes across time (*P*<0.01), there was a significant group × visit interaction (*P*=0.01). Pairwise comparisons revealed that both groups showed a significant reduction in smoking rate from baseline to week 2 (*P*<0.001). However, the galantamine group exhibited a reduction of 2.3 cigarettes per day (±0.32, *d*=0.38) whereas the placebo group only reduced by 1.3 cigarettes per day (±0.30, *d*=0.19). This corresponds to a 12% reduction for the galantamine group and a 7% reduction for the placebo group. There were no significant changes in CO levels across visits or between treatments (data not shown; *P*-values >0.4).

#### Repeated galantamine treatment in human smokers reduces smoking satisfaction and reward

**Figure 6:**
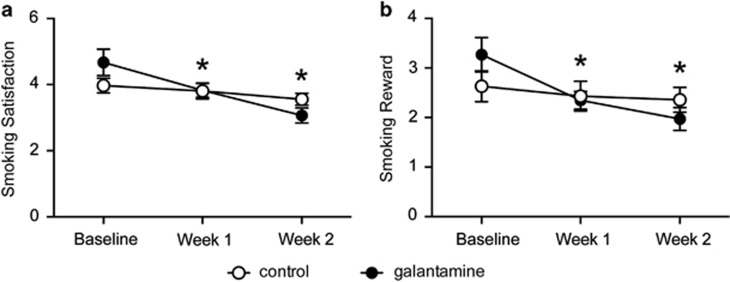
The subjective effects of smoking were significantly reduced in human smokers treated with repeated galantamine. Repeated galantamine treatment significantly reduced smoking satisfaction (**a**) and smoking reward (**b**) during weeks 1 and 2 of treatment (*P<0.001). No significant changes in smoking satisfaction and smoking reward were noted in the placebo group.

Next, we evaluated the effects of galantamine on the subjective effects of smoking during the 2-week pre-quit period while individuals were still smoking. The galantamine group reported marginally higher smoking satisfaction at baseline, compared with placebo group (mean=4.7 (±0.1.5) and 3.9 (±0.95), respectively; *P*=0.07). The groups did not differ in any other subjective measure at baseline (*P*-values >0.14). There were significant group × visit interactions on smoking satisfaction (*P*=0.003) and reward (*P*=0.01). The galantamine group reported significant reductions in smoking satisfaction from baseline to week 2 (*P*<0.001, *d*=1.3; Figure 6a). Likewise, the galantamine group reported that smoking was less rewarding at week 2, compared with baseline (*P*<0.001, *d*=1.1; Figure 6b). The placebo group reported no changes in satisfaction or reward at either visit (*P*-values >0.3, *d*-values <0.48). There were no significant group × visit interactions for craving (*P*=0.07), withdrawal (*P*=0.4), positive affect (*P*=0.44) or negative affect (*P*=0.5, data not shown).

#### Repeated galantamine treatment in human smokers is associated with minimal side effects

**Figure 7:**
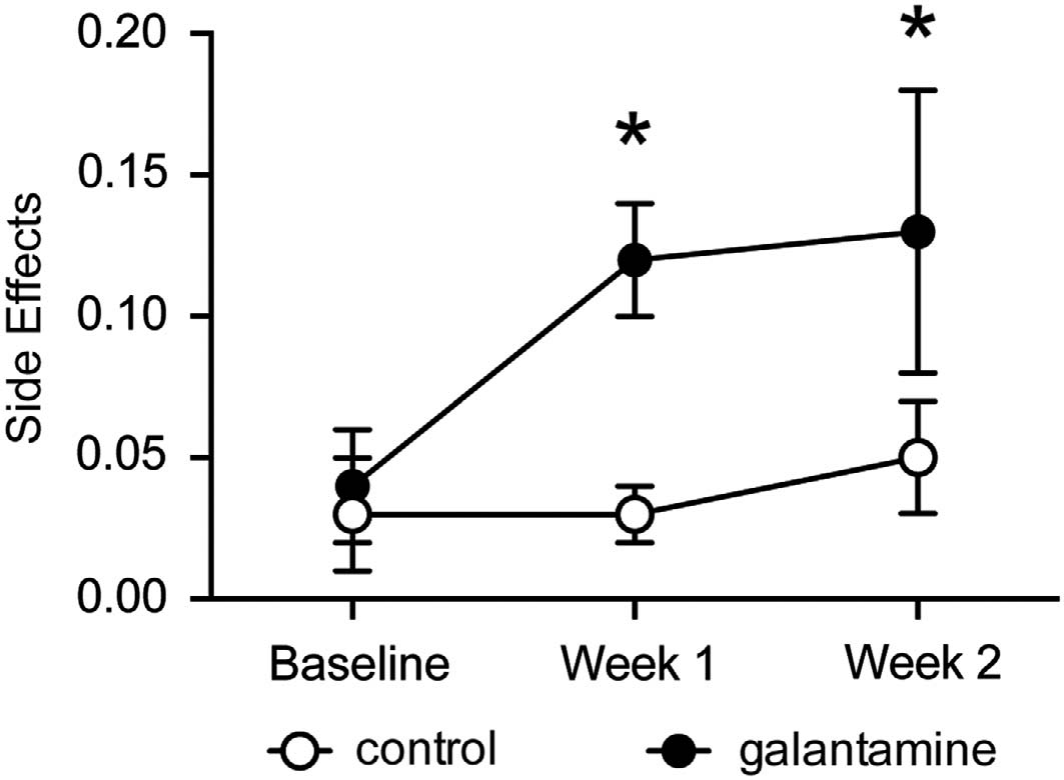
Repeated galantamine treatment in human smokers is associated with minimal adverse effects. Smokers treated with galantamine reported significantly more side effects that placebo-treated controls (*P<0.05). However, these adverse effects were rated as mild and did not preclude smokers from continuing with the treatment.

There were no group differences in side effects at baseline (*P*=0.8; Figure 7). The galantamine group reported an increase in the side effects from baseline to week 2 (*P*=0.01). However, the overall group × visit interaction was not significant (*P*=0.07; Figure 7). Because pica was used to measure nausea/malaise in rodents, nausea was examined separately. At baseline, neither group endorsed symptoms of nausea. At week 1, 11% (*n*=2) and 33% (*n*=5) of the placebo and galantamine groups endorsed nausea, respectively (*χ*^2^=2.4, *P*=0.12). At week 2, 17% (*n*=3) and 27% (*n*=4) of the placebo and galantamine groups endorsed nausea, respectively (*χ*^2^=0.49, *P*=0.48). Overall, the most commonly reported side effects at week 2 were nausea (21%), headache (15%), diarrhea (15%) and heartburn (15%), and did not differ by group (*P*-values >0.11). Except for two participants in the galantamine group who reported moderate nausea and one who reported moderate headache at the week 2 visit, all the symptoms were rated as mild. No participants discontinued the study because of side effects.

Because the galantamine group reported more side effects compared with the placebo group, we wanted to rule out the possibility that the observed decreases in smoking behavior and subjective effects of smoking were not due to increased adverse effects of medication. We conducted an exploratory set of analyses including the change in the side effects summary score from baseline to week 2 as a covariate. Side effects were unrelated to cigarettes per day (*P*=0.3), smoking satisfaction (*P*=0.8) or reward (*P*=0.28) and the group × visit interactions for all the models remained significant (*P*-values <0.01).

## Discussion

To the best of our knowledge, this is the first translational study to demonstrate that repeated AChEI administration decreases nicotine taking in both rats and human smokers. Here, we show that repeated administration of the AChEIs galantamine and donepezil dose-dependently attenuated nicotine, but not sucrose, self-administration in rats. Moreover, repeated AChEI administration did not produce pica or alter *ad libitum* food intake and body weight in rats. Taken together, these results indicate that repeated AChEI administration reduces nicotine consumption in rats and that these effects are not owing to drug-induced nausea/malaise or general motor-suppressant effects of drug treatment in this rat model. Translating these preclinical findings to humans, we found that repeated galantamine treatment significantly reduced total cigarettes smoked per day, smoking satisfaction and smoking reward compared with placebo. Although repeated galantamine was associated with increased side effects (most notably nausea) compared with placebo, these effects were relatively mild and did not limit further participation in the trial. Although the therapeutic benefit of AChEI treatment in healthy human smokers needs to be explored in a larger clinical trial, our provocative findings indicate that AChEIs could be repurposed for smoking cessation.

The present findings are consistent with and expand upon previous studies of acute AChEI administration on nicotine self-administration in rats^10, 11, 38^ and indicate that increased cholinergic transmission is sufficient to reduce nicotine reinforcement. Both acute^10^ and repeated (present study) administration of 5.0 mg kg^−1^ galantamine reduced nicotine taking. Interestingly, while acute administration of 1.0 and 3.0 mg kg^−1^ donepezil reduced nicotine self-administration,^39^ repeated 3.0 mg kg^−1^ donepezil maintained reduced nicotine-taking behavior over 10 days (present study). Despite the initial decrease in nicotine self-administration on day 1 in rats pretreated with 1.0 mg kg^−1^ donepezil in the present study, tolerance developed with repeated administration. The mechanisms underlying tolerance to repeated 1.0 mg kg^−1^ donepezil remain unclear and warrant further investigation. These preclinical findings are largely consistent with our clinical data in human smokers. Here, we show for the first time that repeated galantamine treatment significantly reduced smoking behavior compared with placebo in healthy human smokers without comorbidities. Importantly, reducing smoking behavior before a quit attempt may improve abstinence rates.^40, 41^ Indeed, even a 1% reduction in daily cigarettes smoked over 6 weeks increased the odds of abstinence at 24 weeks by 3%, suggesting that even a small reduction in smoking behavior may be clinically relevant.^42^ Our data indicate that galantamine reduced cigarettes per day by 12% over the 2-week treatment period compared with a 7% reduction in the placebo group. In contrast, repeated donepezil had no effect on the smoking behavior.^16^ However, this study used a dose of donepezil (5 mg) lower than the typical dose (10 mg) prescribed for treating cognitive deficits.^5^ Thus, it is plausible that higher doses of donepezil would reduce smoking behavior consistent with the effects of the clinically effective dose of galantamine used in the present study.

The precise neural mechanisms underlying the effects of AChEIs on nicotine taking are unclear. Donepezil functions solely as a pharmacological inhibitor of acetylcholinesterase,^43^ whereas galantamine also acts as a positive allosteric modulator of nicotinic acetylcholine receptors.^44, 45^ β2-containing nicotinic acetylcholine receptors (nAChRs) have a critical role in nicotine dependence^46, 47^ and recent evidence indicates that acute administration of a positive allosteric modulator of α4β2* nAChRs reduced voluntary nicotine taking in rats.^38^ Therefore, it is likely that the effects of galantamine on nicotine self-administration are because of increased acetylcholine levels and enhanced activation of α4β2* nAChRs in the brain. Galantamine's pharmacological profile may also explain why lower doses of donepezil had no effect on smoking behavior in our prior study.^16^ Future studies are required to identify the precise cholinergic mechanisms and nAChR subtypes that mediate the effects of AChEIs on nicotine self-administration.

Decreased responding for nicotine in rats pretreated with donepezil and galantamine could reflect a decrease in the reinforcing efficacy of nicotine and/or increased drug satiation analogous to higher nicotine doses. This hypothesis is supported by our clinical findings. In human smokers, we found that galantamine significantly reduced self-reported smoking satisfaction and reward when compared with placebo. Similarly, galantamine has been found to attenuate the subjective effects of intravenous nicotine in abstinent smokers.^17^ The subjective effects of drugs of abuse, including nicotine, can be studied using the drug-discrimination paradigm in both rats and humans. The interoceptive effects of nicotine serve as discriminative stimuli to indicate how to obtain a reinforcer (usually lever pressing for a food pellet). By measuring discrimination between operant manipulanda (for example, lever pressing), the ability of drugs to substitute for or modulate the subjective effects of nicotine can be assessed.^48^ The ability of galantamine to reduce the subjective effects of nicotine in our human study may be partially explained by evidence indicating that AChEIs substitute for the discriminative stimulus properties of nicotine in rats.^49^ Galantamine may attenuate nicotine taking, in part, by producing subjective effects similar to nicotine.^49^ The interoceptive properties of nicotine are mediated primarily by α4β2* nAChRs.^50, 51^ Positive allosteric modulators of α4β2* nAChRs, when combined with a sub-threshold dose of nicotine, produce nicotine-like discriminative stimulus effects.^52^ Moreover, the α4β2* nAChR partial agonist varenicline also generalizes to the discriminative stimulus properties of nicotine^53, 54^ and attenuates nicotine taking in rats.^55, 56^ Thus, it is possible that AChEIs may increase or enhance the interoceptive cues of nicotine by activating α4β2* nAChRs thereby reducing subsequent nicotine consumption. These findings are clinically relevant as higher levels of smoking satisfaction and hedonic reactivity to cigarettes predict smoking relapse.^57, 58^

The current study has several limitations. The clinical findings in human smokers are preliminary and should be interpreted with caution. For instance, the baseline difference in smoking satisfaction may have contributed to the observed treatment × time interaction for this subjective measure. However, the galantamine group, but not the placebo group, exhibited an additional decrease in smoking satisfaction between week 1 and week 2. These results suggest that the significant reduction in smoking satisfaction was likely owing to treatment and not baseline differences in this subjective measure. As we focused this study on the pre-quit period while smokers were smoking *ad libitum*, it was not surprising that no changes were observed for craving, withdrawal or mood. The effects of galantamine on withdrawal, craving and mood during abstinence should be tested in future clinical trials. Despite the fact that the galantamine group exhibited a significant reduction in smoking rate, there was not a corresponding decrease in breath CO. Participants were smoking *ad libitum* and it is likely that the variability in the time since last cigarette may have reduced our ability to detect subtle changes in CO.^59^ The lack of biochemical verification limits the conclusions that can be drawn about potential efficacy of galantamine in human smokers. Future studies should consider incorporating biochemical measures that may be more sensitive to changes in smoking rate, such as salivary cotinine. Another important consideration with regard to the present study is the dosing regimen, which was 2 weeks shorter than typically prescribed in patients with cognitive deficits.^60^ Therefore, it is possible that 4 weeks of galantamine would have yielded larger effects. Nevertheless, the effect sizes for galantamine on the smoking rate and smoking satisfaction ranged from *d*=0.38 to 1.3. In addition, galantamine increased side effects, namely nausea, relative to placebo. Although the current sample size was not large enough to formally test mediation, our exploratory analyses indicated that side effects (including nausea) were unrelated to smoking behavior or subjective effects and did not account for the effects of galantamine observed in the current study.

## Conclusions

The present findings provide the first evidence that AChEIs could be repurposed for smoking cessation in human smokers without comorbidities and highlight the importance of translational research for identifying and evaluating novel therapeutic targets for treating nicotine dependence. The consistency between our preclinical and clinical findings provides compelling evidence that a larger clinical trial testing the efficacy of AChEIs for smoking cessation is warranted. Future studies should also focus on potential mechanisms, such as adverse effects associated with smoking withdrawal. AChEIs are cognitive enhancers that have pro-cognitive effects in healthy smokers^16^ and AChEI administration reverses nicotine withdrawal-induced cognitive impairments in mice.^61, 62^ As cognitive deficits predict smoking relapse,^63, 64^ improving cognitive performance during early withdrawal may enhance smoking cessation rates.^6, 7^ Our future studies will evaluate whether AChEIs attenuate withdrawal-related cognitive deficits and improve short-term abstinence using a well-validated medication-screening paradigm.^65, 66^
