# Repeated administration of an acetylcholinesterase inhibitor attenuates nicotine taking in rats and smoking behavior in human smokers

**DOI:** 10.1038/tp.2017.46

**Published:** 2017-03-28

**Authors:** R L Ashare, B A Kimmey, L E Rupprecht, M E Bowers, M R Hayes, H D Schmidt

**Correction to**: *Translational Psychiatry* (2016) **6**, e713; doi:10.1038/tp.2015.209; published online 19 January 2016

The authors noticed that an omission was made in the Acknowledgements section. The omitted data appear below.

This project was partially funded by a grant from the Pennsylvania Department of Health.

