# Supplementary material for: Repeated administration of an acetylcholinesterase inhibitor attenuates nicotine taking in rats and smoking behavior in human smokers
Source: Transl Psychiatry. 2016 Jan 19;6(1):e713–. doi: 10.1038/tp.2015.209 (PMC5068882; doi:10.1038/tp.2015.209)
Supplement: Supplementary Information [file tp2015209x1.doc]

**Supplementary Materials and Methods**

*Rat Studies:*

*Animals and Housing:* Male Sprague Dawley rats (*Rattus norvegicus*) weighing 225-250 g were obtained from Taconic Laboratories (Germantown, NY, USA). Initially, animals were single-housed with access to standard lab chow and water *ad libitum*. Rats used for nicotine and sucrose self-administration studies were mildly food restricted (~20 g chow daily) to 85-90% of their free-feeding body weight following recovery from surgery. Mild food restriction was used to facilitate acquisition and maintenance of nicotine self-administration similar to previously published reports (Corrigall and Coen, 1989; Fowl*er et* al, 2011; L*ee et* al, 2014). Food intake, pica and bodyweight experiments were conducted in separate cohorts of animals that were maintained on *ad libitum* access to chow, except as noted below. All animals were housed in a colony maintained on a 12-h/12-h reverse light/dark cycle, with lights off at 7:00 a.m. All experimental procedures were conducted during the dark phase of the light/dark cycle. All experimental protocols were in accordance with the guidelines set forth by the National Institutes of Health and were approved by the University of Pennsylvania School of Medicine Institutional Animal Care and Use Committee.

*Materials:* All self-administration experiments were conducted in ventilated, sound attenuating operant chambers purchased from Med-Associates Inc. (East Fairfield, VT, USA). Each operant chamber was equipped with both active and inactive response levers, a sucrose pellet dispenser, cue lights, tone generator, as well as an automated injection pump for administering drug or vehicle solutions intravenously.

*Surgery:* Rats were handled daily and allowed one week to acclimate to their home cages upon arrival. Prior to surgery, the rats were anesthetized with 80 mg/kg ketamine and 12 mg/kg xylazine (Sigma Aldrich/RBI, St. Louis, MO, USA). An indwelling silastic catheter (CamCaths; Cambridge, UK) was inserted into the right, external jugular vein and sutured securely in place. The catheter was connected to a mesh backmount, which was implanted subcutaneously above the shoulder blades. To prevent infection and maintain patency, catheters were flushed daily with 0.3 ml of a solution of the antibiotic Timentin (0.93 mg/ml; Fisher, Pittsburgh, PA, USA) dissolved in heparinized 0.9% saline (Butler Schein, Dublin, OH, USA). When not in use, catheters were sealed with plastic obturators.

*Nicotine Self-Administration:* Rats were allowed seven days to recover from surgery before behavioral testing commenced. Initially, rats were placed in operant chambers and allowed to lever press for intravenous nicotine (0.03 mg/kg nicotine/59 μl saline, infused over 5 s) on a fixed-ratio 1 (FR1) schedule of reinforcement. Each nicotine infusion was paired with a light/tone cue. Stable responding on the FR schedules of reinforcement was defined as less than 20% variation in response rates over three consecutive self-administration days. After stable responding was achieved, the schedule of reinforcement was increased to fixed-ratio 3 (FR3) for 3-5 days and then finally increased to a fixed-ratio 5 (FR5) schedule. For all FR schedules, a 20 second time-out period followed each nicotine infusion, during which time active lever responses were recorded but had no scheduled consequences. Responses made on the inactive lever, which had no scheduled consequences, were also recorded during each 2-h daily self-administration session and were used as a measure of nonspecific motor effects of drug treatment.

*Sucrose Self-Administration:* Rats were trained initially to lever press for 45 mg sucrose pellets (Research Diets, Inc., New Brunswick, NJ, USA) on a FR1 schedule of reinforcement. Once animals achieved stable responding for sucrose (defined as less than 20% variation in responding over three consecutive sessions) on the FR1 schedule of reinforcement, the response requirement was increased to a FR5 schedule of reinforcement. Animals were limited to 30 sucrose pellets within a 1-hour operant session and were restricted to 20 g of lab chow daily (Harlan Teklad, Wilmington, DE, USA) in their home cages for the duration of the experiment. Rats were mildly food restricted in order to maintain consistency with the nicotine self-administration experiments (i.e., to ensure similar motivational states). Each successful completion of the response requirement resulted in delivery of a sucrose pellet as well as contingent presentation of light/tone cues.

*Pica and Ad Libitum Food Intake:* Separate cohorts of rats were single-housed on a 12-h/12-h reverse light/dark cycle with the lights off at 9:00 a.m. Consistent with the aforementioned studies of galantamine and donepezil on nicotine and sucrose self-administration, all experimental procedures were performed during the dark phase of the light/dark cycle. Initially, rats were habituated to *ad libitum* kaolin (Research Diets; K50001) access for one week while maintained on standard rodent chow. Baseline kaolin intake during the habituation phase was negligible (mean kaolin intake <0.5 g for 24 h; data not shown). Prior to experimental testing, rats were mildly food restricted from 16:00h to 09:00h, with the majority of food restriction occurring during the rats’ light cycle to ensure an acute, energy depleted state similar to the chronic deprivation state of rats used in the nicotine self-administration studies.

*Human Studies:*

*Participants:*  Exclusion criteria included: pregnancy, lactation, or planning pregnancy; positive urine drug screen; low or borderline intelligence (Shipley IQ score < 90) (Zachary, 2000); consumption of more than 25 alcoholic drinks per week; heart attack or cancer in previous 6 months; lifetime history of stroke; medical conditions contraindicated with galantamine (e.g., peptic ulcer disease; kidney or liver failure); uncontrolled hypertension; Alzheimer’s disease diagnosis; seizure disorder; current diagnosis or history of DSM IV Axis 1 disorders (except nicotine dependence); current diagnosis of major depression; and current use of smoking cessation treatment, psychotropic medications, or contraindicated medications.

*Study Design and Procedures*: This human laboratory study adapts a well-validated medication screening paradigm for short-term smoking abstinence (Perki*ns et* al, 2010; Perki*ns et* al, 2008). The study employed a two week run-up procedure in which smokers were treated with placebo or 8mg galantamine (q.d.) daily for the first week and placebo or 16mg galantamine (q.d.) during the second week. Following this 2-week run-up period, participants attended a 1.5 visit (week 2) during which they completed the same measures as the baseline visit in addition to a 20-min coaching session to prepare them for the 24h abstinence period. After abstaining from smoking for 24h, participants completed another testing session similar to the week 2 visit followed by a programmed lapse cigarette. The participants finished the study with four observation visits during a 7-day quit week during which they were asked to try their best to abstain from smoking. In the present manuscript only data from the baseline, week 1, and week 2 visits are reported.

| **Table S1. Human Study: Demographic and smoking characteristics by group (total *N* = 33)** | | | | |
| --- | --- | --- | --- | --- |
|  | **Group** | | |  |
|  | **Placebo**  **(*n* = 18)** |  | **Galantamine**  **(*n* = 15)** | ***p-*value** |
| Sex (n, % female) | 5, 28% |  | 8, 53% | 0.14 |
| Age (years) | 41.5 (11) |  | 44.9 (10) | 0.36 |
| Race (n, % Caucasian) | 4, 22% |  | 4, 26% | 0.97 |
| Shipley IQ score | 104.5 (8) |  | 102.9 (7) | 0.54 |
| BMI | 29.8 (6) |  | 30.0 (6.7) | 0.90 |
| Nicotine dependence | 4.8 (2.3) |  | 4.9 (1.7) | 0.89 |
| Baseline cigarettes per day | 14.2 (4.9) |  | 15.4 (4.4) | 0.48 |
| Years smoking | 22.8 (11) |  | 28.0 (9) | 0.16 |
| CO at Intake (ppm) | 22.4 (9) |  | 19.3 (8) | 0.30 |
| *Notes*: Values are mean (standard deviation) unless otherwise noted. | | | | |

**References**

Corrigall WA, Coen KM (1989). Nicotine maintains robust self-administration in rats on a limited-access schedule. *Psychopharmacology (Berl)* **99**(4): 473-478.

Fowler CD, Lu Q, Johnson PM, Marks MJ, Kenny PJ (2011). Habenular alpha5 nicotinic receptor subunit signalling controls nicotine intake. *Nature*.

Lee AM, Arreola AC, Kimmey BA, Schmidt HD (2014). Administration of the nicotinic acetylcholine receptor agonists ABT-089 and ABT-107 attenuates the reinstatement of nicotine-seeking behavior in rats. *Behav Brain Res* **274**: 168-175.

Perkins KA, Lerman C, Fonte CA, Mercincavage M, Stitzer ML, Chengappa KN*, et al* (2010). Cross-validation of a new procedure for early screening of smoking cessation medications in humans. *Clin Pharmacol Ther* **88**(1): 109-114.

Perkins KA, Lerman C, Stitzer M, Fonte CA, Briski JL, Scott JA*, et al* (2008). Development of procedures for early screening of smoking cessation medications in humans. *Clin Pharmacol Ther* **84**(2): 216-221.

Zachary RS (2000). *Shipley Institute of Living Scale - Revised Manual* Western Psychological Services.
